# Cathepsin B pH-Dependent Activity Is Involved in Lysosomal Dysregulation in Atrophic Age-Related Macular Degeneration

**DOI:** 10.1155/2019/5637075

**Published:** 2019-12-06

**Authors:** Audrey Voisin, Christelle Monville, Alexandra Plancheron, Emile Béré, Afsaneh Gaillard, Nicolas Leveziel

**Affiliations:** ^1^University of Poitiers, Laboratoire de Neurosciences Expérimentales et Cliniques, Equipe Thérapie Cellulaire dans les Pathologies Cérébrales, Poitiers F-86073, France; ^2^INSERM, U1084, Laboratoire de Neurosciences Expérimentales et Cliniques, Equipe Thérapie Cellulaire dans les Pathologies Cérébrales, Poitiers F-86022, France; ^3^CHU Poitiers, Poitiers F-86021, France; ^4^INSERM, UMR861, I-Stem, AFM, Genopole Campus 1, Evry F-91030, France; ^5^UEVE-Paris Saclay, UMR861, I-Stem, AFM, CRCT, Corbeil-Essonnes F-91100, France; ^6^CECS/I-Stem AFM, CRCT, Corbeil-Essonnes F-91100, France; ^7^Plateforme ImageUP, 1 Rue Georges Bonnet, F-86022 Poitiers, France

## Abstract

Age-related macular degeneration (AMD) is characterized by retinal pigment epithelial (RPE) cell dysfunction beginning at early stages of the disease. The lack of an appropriate *in vitro* model is a major limitation in understanding the mechanisms leading to the occurrence of AMD. This study compared human-induced pluripotent stem cell- (hiPSC-) RPE cells derived from atrophic AMD patients (77 y/o ± 7) to hiPSC-RPE cells derived from healthy elderly individuals with no drusen or pigmentary alteration (62.5 y/o ± 17.5). Control and AMD hiPSC-RPE cell lines were characterized by immunofluorescence, flow cytometry, and electronic microscopy. The toxicity level of iron after Fe-NTA treatment was evaluated by an MTT test and by the detection of dichloro-dihydro-fluorescein diacetate. Twelve hiPSC-RPE cell lines (6 AMD and 6 controls) were used for the experiment. Under basal conditions, all hiPSC-RPE cells expressed a phenotypic profile of senescent cells with rounded mitochondria at passage 2. However, the treatment with Fe-NTA induced higher reactive oxygen species production and cell death in hiPSC-RPE AMD cells than in hiPSC-RPE Control cells. Interestingly, functional analysis showed differences in lysosomal activity between the two populations. Indeed, Cathepsin B activity was higher in hiPSC-RPE AMD cells compared to hiPSC-RPE Control cells in basal condition and link to a pH more acidic in this cell population. Moreover, oxidative stress exposure leads to an increase of Cathepsin D immature form levels in both populations, but in a higher proportion in hiPSC-RPE AMD cells. These findings could demonstrate that hiPSC-RPE AMD cells have a typical disease phenotype compared to hiPSC-RPE Control cells.

## 1. Introduction

Age-related macular degeneration (AMD), a multifactorial disease caused by age and genetic and environmental factors [1], is the first cause of blindness in the elderly population in developed countries [2]. The disease is characterized by the accumulation of drusen, extracellular deposits of proteins and lipids and by progressive cellular degeneration of retinal pigment epithelial (RPE) cells located in the macular area [3]. The exudative form of AMD is characterized by choroidal neovascularization, and the atrophic form, also called dry form, is characterized by progressive RPE cell degeneration finally associated with photoreceptor loss [3]. Understanding the molecular mechanisms involved in AMD has been challenging due to the lack of an appropriate *in vitro* model [4].

Induced pluripotent stem cells (iPSC) derived from somatic cell lines are indistinguishable from embryonic stem (ES) cells in terms of morphology, proliferation, gene expression, and teratoma formation [5]. They also have the ability to be expanded indefinitely in culture and to differentiate into multiple lineages [6]. Many improvements in cell reprogramming and differentiation have yielded specific populations of diversified kinds of cells such as retinal cells [7, 8]. Since the last decade, the generation of RPE cells from hiPSC has been investigated to model the ocular disorders associated with dysfunction of RPE cells [9]. While the ARPE-19, an immortalized human RPE cell line, is currently used as an *in vitro* model for retinal diseases, many studies have reported major differences (pigmentation, RPE cell marker expression, transepithelial resistance, protein secretion level, and so on) between ARPE-19 cells and human fetal or adult hRPE cells and iPSC-RPE cells [10–12].

RPE cells are highly polarized monolayer cells characterized by pigmentation, octagonal morphology, and tight junction. These cells play a key role in many functions such as retinal blood barrier, nutriment and water input, light absorption and phagocytosis of photoreceptor outer segment (POS), and retinol recycling [13, 14]. Many studies have observed morphological and functional changes in RPE cells during the aging process (mitochondrial damage, lysosomal dysregulation, accumulation of lipofuscin, and so on) suggesting that these cells play a role in the pathogenesis of AMD [15–17]. Chronic oxidative stress is likely an important contributing environmental risk factor to the development of AMD. Previous *in vitro* studies have shown that exposure to drugs inducing oxidative stress leads to both functional and morphological RPE alterations [18, 19]. Indeed, accumulation of iron, an essential element in many metabolic processes that accumulates with normal aging [16], may be involved in the pathogenesis of AMD as a source of free radicals contributing to tissue damage through lipidic membrane alterations and protein modifications [20]. Iron is responsible for reactive oxygen species (ROS) production by Fenton reaction, and it has been observed that iron accumulates more within the macular area and RPE cells in people affected by atrophic AMD [21]. One consequence of RPE cell oxidative stress exposure is the rapid formation and accumulation of nondegradable pigment lipofuscin within the lysosomal compartment hampering phagocytosis and eventually promoting cell death [22]. Dysregulation of autophagy, a lysosome-mediated degradation process for nonessential or damaged cellular constituents, seems to have a role in AMD development [23].

This study is aimed at comparing lysosomal function of hiPSC-RPE cells derived from healthy individuals to those derived from patients affected with atrophic AMD under oxidative stress conditions induced by iron intracellular accumulation. Indeed, it has been shown recently that iPSC-RPE cells derived from both skin and RPE cells of AMD donors exhibit AMD-like phenotypes including susceptibility to oxidative stress, increased levels of ROS, and lower mitochondrial activity [4, 24]. In our study, we demonstrated that hiPSC-RPE cells derived from patients affected by atrophic AMD expressed a specific disease phenotype compared to iPSC-RPE cells derived from healthy individuals. We confirmed that hiPSC-RPE cells derived from AMD patients could be a useful tool to study pathological mechanisms leading to the development of AMD or to evaluate potential therapeutic molecules.

## 2. Materials and Methods

### 2.1. Patients

Peripheral venous blood samples from individuals without retinal disease and patients affected by atrophic AMD were obtained after obtaining informed consent and in accordance with the Committee for Protection of Persons (no. 20152528) and with the tenets of the Declaration of Helsinki. Fibroblast cell lines were obtained from Coriell Institute for Medical Research (New Jersey, USA).

Retinal phenotype of atrophic AMD patients was assessed by multimodal imaging including color fundus photographs and OCT (Optical Coherence Tomography) scans (Spectralis®, Heidelberg Engineering, Germany).

### 2.2. hiPSC Generation from Blood Cells and Fibroblasts

We followed the methods of Voisin et al. [12]. Peripheral blood mononuclear cells (PBMC) were isolated from the blood by Ficoll. They were maintained in StemSPAN media (StemCell, Vancouver, Canada) supplemented with 50 ng/mL SCF (R&D, Lille, France), 2 U/mL EPO (R&D), 10 *μ*g/mL FGF2 (Peprotech, Neuilly-sur-Seine, France), 500 U/mL, 1 mM dexamethasone (Sigma), 40 *μ*g/mL IGF-1 (Miltenyi, Bergisch Gladbach, Germany), 10 *μ*g/mL IL-3 (Miltenyi), and 50 ng/mL ascorbic acid (Sigma). Cells were nucleofected with Yamanaka factors OCT3/4, Sox2, KLF4, and L-Myc (Addgene, Cambridge, MA, USA: pCXLE-hOCT3/4-shp53-F, pCXLE-hUL, and pCXLE-hSK). At day 4 after nucleofection, cells were plated onto feeders (MTI-GlobalStem, Gaithersburg, MD, USA). During 10 days, cells were cultured into DMEM/F12 media supplemented with 10% KSR (Invitrogen, Carlsbad, CA, USA), 10 *μ*g/mL FGF2, 2 *μ*M SB431542 (R&D), 0.5 *μ*M PD0325901 (Euromedex, Souffelweyersheim, France), 2 *μ*M thiazovivin (R&D), 3 *μ*M CHIR99021 (Bertin Pharma, Montigny-le-Bretonneux, France), 0.5 mM VPA (Sigma), and 0.25 mM NaB (Sigma).

Fibroblast cell lines were nucleofected with Yamanaka factors using the same protocol as described for PBMC. At day 4 after nucleofection, DMEM/F12 media were supplemented with 10% KSR, 10 *μ*g/mL FGF2, 2 *μ*M SB431542, 0.5 *μ*M PD0325901, and 0.5 mM VPA.

At day 10 postreprogramming, VPA and NaB were removed from the culture medium. At day 15 postreprogramming, SB461542, PD0325901, CHIR99021, and thiazovivin were removed from the culture medium. hiPSC colonies appeared after around 15-20 days of culture and were collected about 2-3 weeks postnucleofection. The culture medium (DMEM F/12+20% KSR+10 *μ*g/mL FGF2) was changed every day.

### 2.3. Differentiation of hiPSC into RPE

hiPSC were differentiated into RPE cells by switching the culture medium to DMEM High Glucose (Invitrogen) supplemented with 20% KSR without FGF2 as previously described [7, 12]. Pigmented areas with typical RPE appearance usually appeared three weeks after initiation of differentiation. Specific selection of RPE cells was obtained after manual transfer of the pigmented colonies into new plates (P0). hiPSC-RPE cells were cultured with DMEM High Glucose supplemented with 4% KSR. The medium was changed twice a week. hiPSC-RPE cells were passaged every 3 weeks up to two passages.

### 2.4. hiPSC and RPE Characterization

As previously described [12], cells were stained with pluripotency and RPE-specific cell markers for immunostaining and flow cytometry analysis. Briefly, hiPSC were fixed with cold 4% paraformaldehyde for 20 min and washed twice with phosphate-buffered saline (PBS). Nonspecific binding sites and permeabilization were performed using a blocking solution containing PBS, 0.1% Triton X-100 (Sigma), 5% Goat Serum, and 5% Horse Serum (Sigma) for 1 hour. Cells were then incubated overnight at 4°C with the following primary antibodies diluted in blocking solution: OCT-4 (1/200, Abcam, Cambridge, MA, USA), NANOG (1/200, Abcam), ZO-1 (1/100, Thermo Fisher, Wathman, MA, USA), Bestrophin-1 (1/100, Novus Biological, Littleton, CO, USA), Pax6 (1/75, Abcam), and Tyrosinase (1/100, Abcam). Cells were washed twice in PBS, followed by staining with the secondary antibodies (for OCT-4, Bestrophin-1, and Tyrosinase: Donkey Anti-Rabbit IgG H&L Alexa Fluor 555 (Abcam); for NANOG, Pax6, and ZO-1: Goat Anti-Mouse IgG H&L Alexa Fluor 488 (Abcam)). Nuclei were stained with DAPI (Invitrogen), and slides were mounted in mowiol (Sigma). Cells were observed with a Zeiss microscope and counted using ZEN software (Iéna, Germany).

For FACS analysis, hiPSC were dissociated using accutase (Thermo Fisher) for 5 min at room temperature, then washed with DMEM/F12 media with 10% KSR and incubated with the following primary conjugated antibodies: anti TRA-1-81-AlexaFluor 647 (1/25; BD Biosciences, Franklin Lakes, NJ, USA) and anti SSEA3-AlexaFluor 488 (1/10; BD Biosciences) for 30 min at 4°C in the dark. Stained cells were washed twice in PBS supplemented with 2% SVF. Cells were analyzed on FACSVerse (BD Biosciences) with 100,000 events acquired for each sample. Data were analyzed with FlowJo® software (Ashland, OR, USA).

For phosphatase alkaline activity, hiPSC were fixed with 95% ethanol and incubated 10 minutes with the Sigma Fast BCIP/NBT kit (Sigma) following the manufacturer's instructions.

### 2.5. Transmission Electron Microscopy

hiPSC-RPE cells were fixed for 1 h with 2.5% glutaraldehyde in 1 M phosphate-buffered saline (pH 7.1). After PBS washes, cells were post-fixed for 45 min in 1% osmium tetroxide in phosphate buffer. Dehydration was carried out using successive incubations of increasing ethanol concentrations (from 50% to 100%). Throughout this experimental protocol, each incubation was centrifuged at 1000 rpm for 5 min at room temperature. Cell pellets were included in Epon resin and after 24 h of polymerization; 70 nm sections were revalidated using an ultracut UC6 of LEICA. Uranyl acetate (2% in 70% ethanol) and lead citrate were used as contrasting agents. Pictures were obtained on a JEOL 1010 electron microscope at 80 kV with an Olympus digital camera (Quemesa) using Item software.

### 2.6. Measure of Senescence by *β*-Gal Activity

hiPSC-RPE cells were seeded in 24-well plates and allowed to mature for up to 3 weeks following final passage (P2). Cells at confluency showing typical cobblestone morphology and displaying pigmentation were stained with SA-*β*-gal assay detection kit (Abcam) according to the manufacturer's instructions. The percentage of SA-*β*-gal positive cells was calculated by counting the number of labeled cells manually with ZEN software.

### 2.7. Iron Treatment and Induction of Oxidative Stress

hiPSC-RPE cells (3 weeks after P2) were treated for 24 hours with Fe-NTA at final concentrations of 5 to 20 mM. Cells were washed with PBS and incubated with 1 mL of fresh culture medium supplemented by 10 *μ*g/mL of DCFH-DA [25] (Sigma) for 1 hour at 37°C. Oxidation of dichloro-dihydro-fluorescein (DCFH) by ROS to 2′,7′-dichlorofluorescein DCF led to the formation of a green fluorescent oxidation product [25]. The cells were washed with PBS and maintained in 1 mL of PBS-2% SVF culture medium. Fluorescence was measured with a microplate reader (Berthold Technologies, Bad Wildbad, Germany; excitation 488 nm, emission 535 nm), and the intensity values were calculated with Mithras software (Berthold Technologies).

### 2.8. MTT Assay

MTT assay (Sigma) was used to reflect cellular viability as previously described [26]. Briefly, after 24 hours of Fe-NTA exposure, the medium was replaced with 0.5 mg/mL MTT. Following 3 h and 30 minutes of incubation at 37°C, cells were lysed with DMSO [27]. Absorbance was then read at 540 nm and 620 nm (background) with a microplate reader (Tecan, Männedorf, Suisse) and analyzed with Magellan software (Tecan).

### 2.9. Flow Cytometry Analysis

The change in mitochondrial transmembrane potential induced by Fe-NTA treatment in iPSC-RPE cell lines was observed using the appropriate fluorescent probe JC-1 (Thermo Fisher) [28]. The potential-sensitive color shift of JC-1 is caused by changes in the concentration of red fluorescent JC-1 aggregates. Mitochondrial depolarization is indicated by a decrease in aggregated JC-1 that emits a red fluorescence. Briefly, cells were labeled with 2 *μ*mol/L of JC-1 for 25 min at 37°C, washed with PBS, and analyzed on the flow cytometer FACSVerse (BD Biosciences) using 488 nm excitation with 530 and 585 nm band pass emission filters. The red/green fluorescence intensity ratio quantified mitochondrial potential using FlowJo® software (Ashland, OR).

Lysosomal activity was observed using the lysosomal activity assay kit (Clinisciences, France). After Fe-NTA treatment, cells were incubated for 1 h with 15 *μ*L of self-quenched substrate in 1 mL of media supplemented with 0.5% of FBS. Cells were washed twice with ice-cold 1X assay buffer and analyzed on the flow cytometer FACSVerse (BD Biosciences).

### 2.10. Western Blot

hiPSC-RPE cells were lysed in the extraction buffer containing 50 mM Tris-HCl pH 7.5, 150 mM NaCl, 1 mM EDTA, 10% NP40, 0.1% de SDS, 12 mM sodium desoxycholate, 1 mM Na3VO4, 1 mM NaF, and 2 mM PMSF. Cells were sonicated for 10 seconds before protein concentration determination by Bradford colorimetric assay. Forty micrograms of proteins was separated on SDS-PAGE and electrotransferred onto polyvinylidene difluoride membranes. The membrane was blocked in 5% nonfat milk and 0.1% Tween 20 in PBS and was then incubated with primary antibodies (Transferrin, Santa Cruz; *β*-actin, Invitrogen; Cathepsin D, GeneTex) at 4°C overnight. The membrane was rinsed with 0.1% Tween 20 in PBS and incubated with secondary antibody for 1 hour. Blots were developed by enhanced chemiluminescence (ECL). Relative band density was determined with ImageJ software (developed by Wayne Rasband, National Institutes of Health, Bethesda, MD; available at http://rsb.info.nih.gov/ij/index.html). *β*-Actin was used as a loading and quality control.

### 2.11. Measure of Cathepsin B Activity

To analyze the activity of Cathepsin B (Cat B), we used SensoLyte 520 Cathepsin B assay Kit (AnaSpec) according to the kit instructions. Briefly, treated hiPSC RPE cells were incubated 10 minutes at RT with the assay buffer before adding 50 *μ*L of 1X Cat B substrate solution into each well. The fluorescence signal (Ex/Em: 490 nm/535 nm) was measured after 30 minutes of enzymatic reaction using a microplate reader and analyzed with Magellan software. Positive (Cat B enzyme) and negative controls (assay buffer) were used to ensure the quality of the test.

### 2.12. Acridine Orange Staining for Investigation of Lysosomal Acidification

Lysosomal stability was assessed by the AO-relocation method. AO exhibits red fluorescence at high concentrations (in intact lysosomes), but green fluorescence at low concentrations (when lysosomal contents diffuse into the cytosol) [29]. hiPSC-RPE cells were loaded with 1 *μ*M acridine orange (AO, Thermo Fisher) for 15 min at 37°C and were harvested with PBS (pH 7.4). Quantitative comparisons were performed in 96-well plates, and the fluorescence (excitation/emission.485/535 nm for monomer, 450/665 nm for dimer) was measured with a microplate reader and analyzed with Magellan software. The ratio of fluorescence of the dimer and oligomer to that of the monomer was calculated. For microscopy observations, samples were observed under a fluorescence microscopy. Lysosomal stability was assessed by red AO-fluorescence, using ZEN software.

## 3. Results

### 3.1. Patients

**Figure 1:**
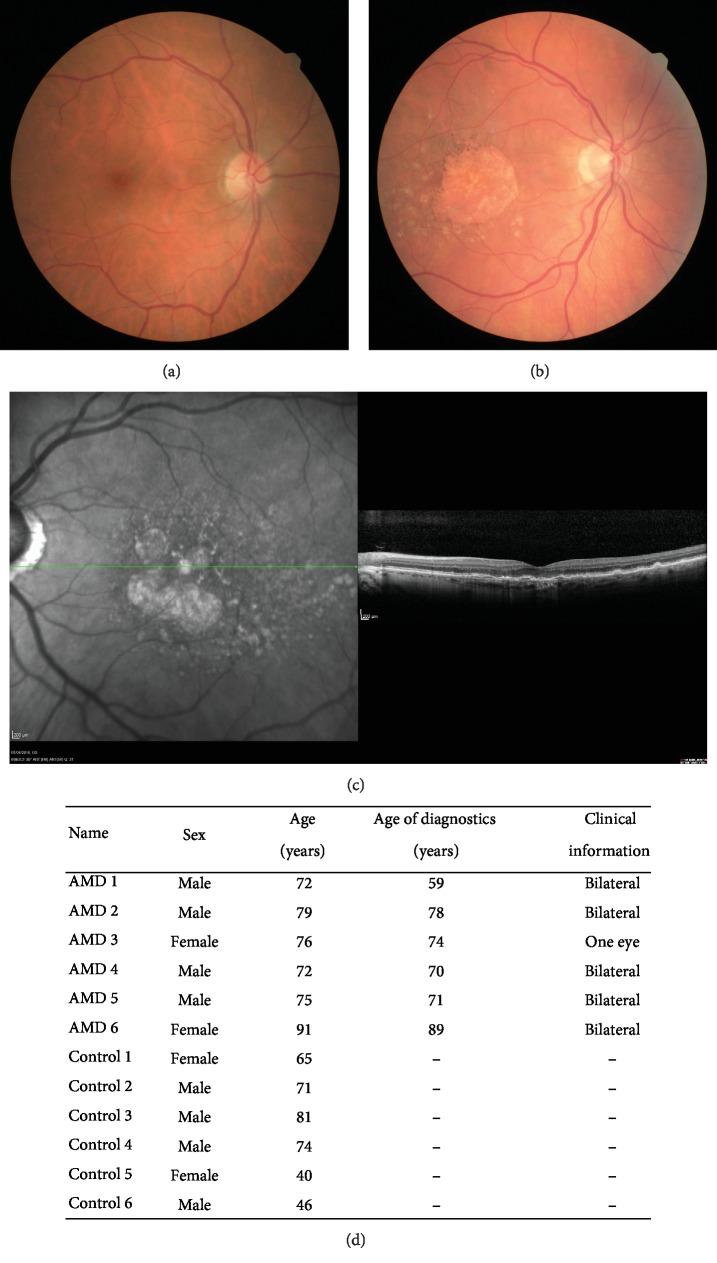
Clinical information on healthy individual and patients affected by atrophic AMD included in this study. Fundus examination of (a) a healthy individual and (b) an atrophic AMD patient. (c) Optical tomography coherence imaging of the patient affected by atrophic AMD. (d) Donor characteristics and clinical information.

Individuals included in the control group (6 patients, 62.8 ± 16 y/o) were patients with normal fundus examination, with no drusen or pigmentary alteration and no familial history of AMD (Figures 1(a) and 1(d)).

The diagnosis of atrophic AMD (6 patients, 77.5 ± 7 y/o) was performed on fundus examination, color photographs, and SD-OCT scans. Patients with any history of exudative AMD or mixt form (atrophic AMD in one eye and exudative AMD in the fellow eye or both forms in the same eye) of the disease were excluded. These patients presented typical accumulation of drusen, RPE, and photoreceptor atrophy and choroidal thinning (Figures 1(b) and 1(c)).

### 3.2. Derivation and Characterization of hiPSC-RPE Cells from Erythroblasts and from Fibroblasts

**Figure 2:**
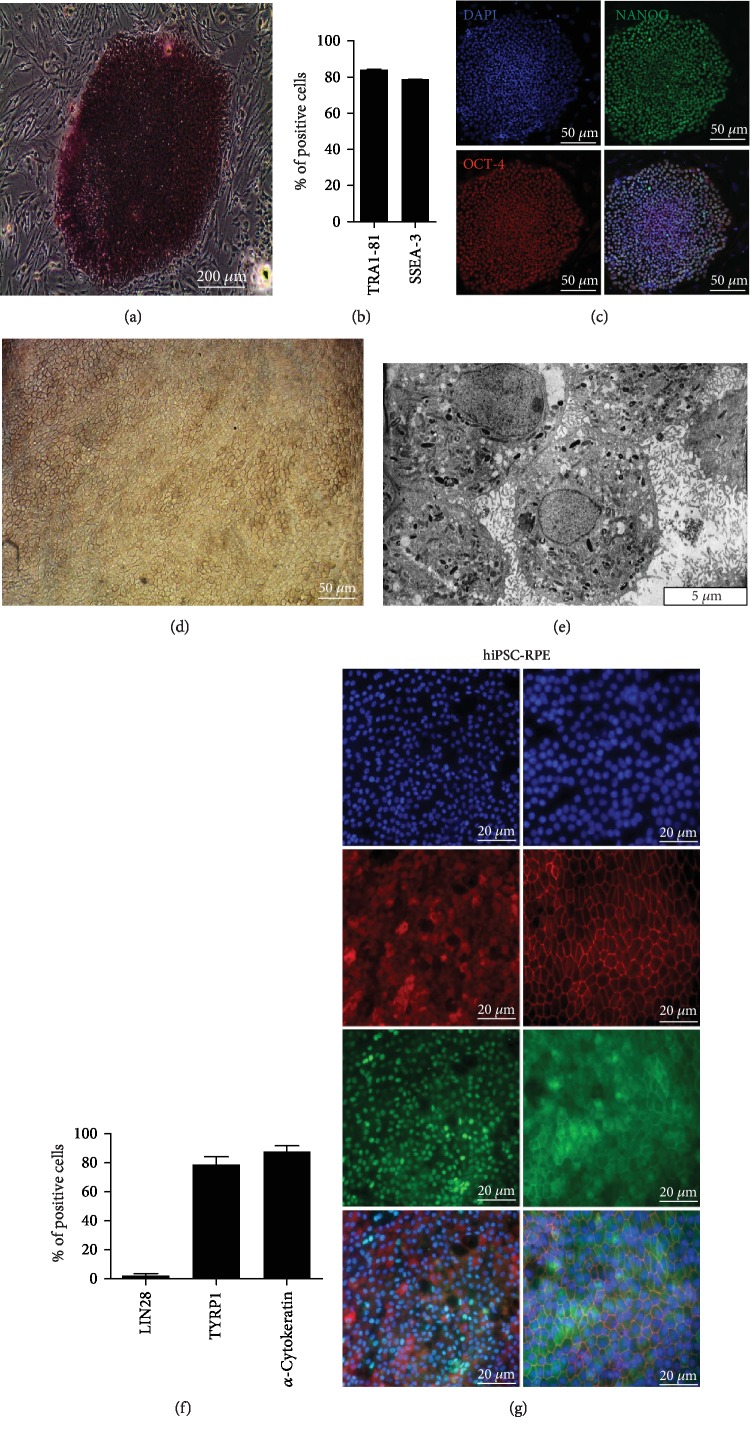
Characterization of hiPSC and hiPSC-RPE cells. (a) Alkaline phosphatase activity of hiPSC. (b) Quantitative flow cytometry analysis of both typical embryonic stem cell surface markers TRA1-81 and SSEA-3. (c) Representative immunofluorescence analysis of pluripotency-specific markers of both stem cells NANOG and OCT-4. Nuclei were stained with DAPI. (d) Typical morphology of hiPSC-RPE cells. (e) *Transmission electron microscopy* analysis of RPE cells showing typical morphologic features such microvilli, mitochondria, and pigmentary granules. (f) Quantitative flow cytometry analysis of stem cell surface marker Lin 28 and of typical RPE cell surface markers TYRP1 and *α*-cytokeratin. (g) Representative immunofluorescence analysis of pluripotency-specific markers of typical RPE cell surface markers BEST-1, PAX6, ZO-1, and TYRP1. Nuclei were stained with DAPI.

hiPSC showed typical hES-like morphology and had alkaline phosphatase activity (Figure 2(a)). Flow cytometry revealed the expression of two key pluripotency markers SSEA-3 and TRA-1-81 (Figure 2(b)). This result was confirmed by immunofluorescence labeling of hiPSC colonies with the expression of two other characteristics markers of stem cells OCT4 and NANOG (Figure 2(c)).

hiPSC-RPE cells formed a confluent monolayer displaying the classical cobblestone morphology (Figure 2(d)). Transmission electronic microscopy showed typical maturation patterns of hiPSC-RPE cells, including microvilli, melanosomes, and mitochondria (Figure 2(e)). Flow cytometry analysis of hiPSC-RPE cells demonstrated the presence of TYRP1 and *α*-cytokeratin and the absence of Lin28, another typical pluripotency marker (Figure 2(f)). This result was confirmed by immunostaining with the expression of RPE-specific marker protein Bestrophin-1 (BEST-1), eye development marker Pax6, tight junction marker zonula occludens 1 (ZO-1), and melanin production marker Tyrosinase (TYRP1) (Figure 2(g)).

### 3.3. Both hiPSC-RPE Control and AMD Cells Have Characteristics Typical of Senescent Cells

**Figure 3:**
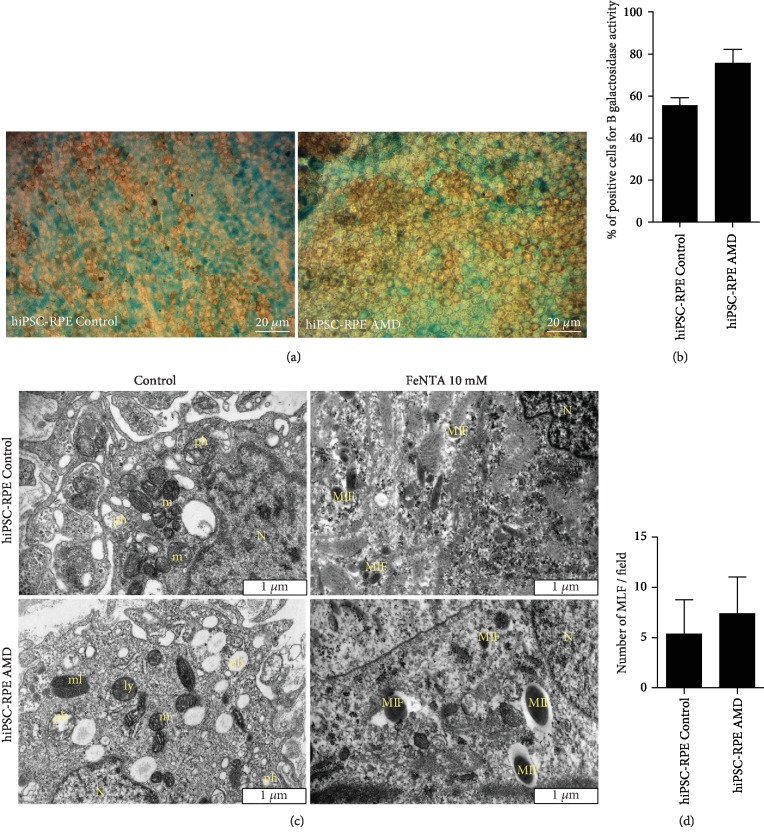
(a) SA-*β*-Gal activity (blue) in both hiPSC-RPE Control and AMD cells. (b) Quantification by manual counting of SA-*β*-Gal activity in both hiPSC-RPE Control cells (*N* = 5) and AMD cells (*N* = 4). Values are the mean ± SEM. hiPSC-RPE Control: 16 451 cell count; hiPSC-RPE AMD: 12 829 cell count. Statistical analysis: *t*-test (two-tailed). (c) Electronic microscopy analysis of hiPSC-RPE Control and AMD cells in both basal and oxidative stress conditions induced by 10 mM Fe-NTA during 24 hours. N: nucleus; m: mitochondria; ml: melanosome; ly: lysosome; pH: phagosome; MLF: melanolipofuscin. (d) Quantification by manual counting of MLF production during 10 mM FeNTA exposure in hiPSC-RPE Control cells (*N* = 3) and AMD cells (*N* = 4).

SA-*β*-Gal (senescence-associated beta-galactosidase) activity is present only in senescent cells and is not found in presenescent, quiescent, or immortal cells [30]. Indeed, in a previous study, we observed that ARPE-19 cell line did not express SA-*β*-Gal activity [12]. Here, we observed SA-*β*-Gal activity in both hiPSC-RPE Control and AMD cells in 49.8 ± 6.3% and 75.7 ± 6.5% of cells, respectively (*p* = 0.0579), suggesting a senescent cell phenotype of these two kinds of population (Figures 3(a) and 3(b)).

Morphological and functional changes of mitochondria occur during aging [31]. In basal condition, both populations of hiPSC-RPE cells showed the rounded mitochondria (Figure 3(c)) to be classically associated with senescent cells [32].

### 3.4. Fe-NTA Exposure Induces More Production of ROS in hiPSC-RPE AMD Cells

**Figure 4:**
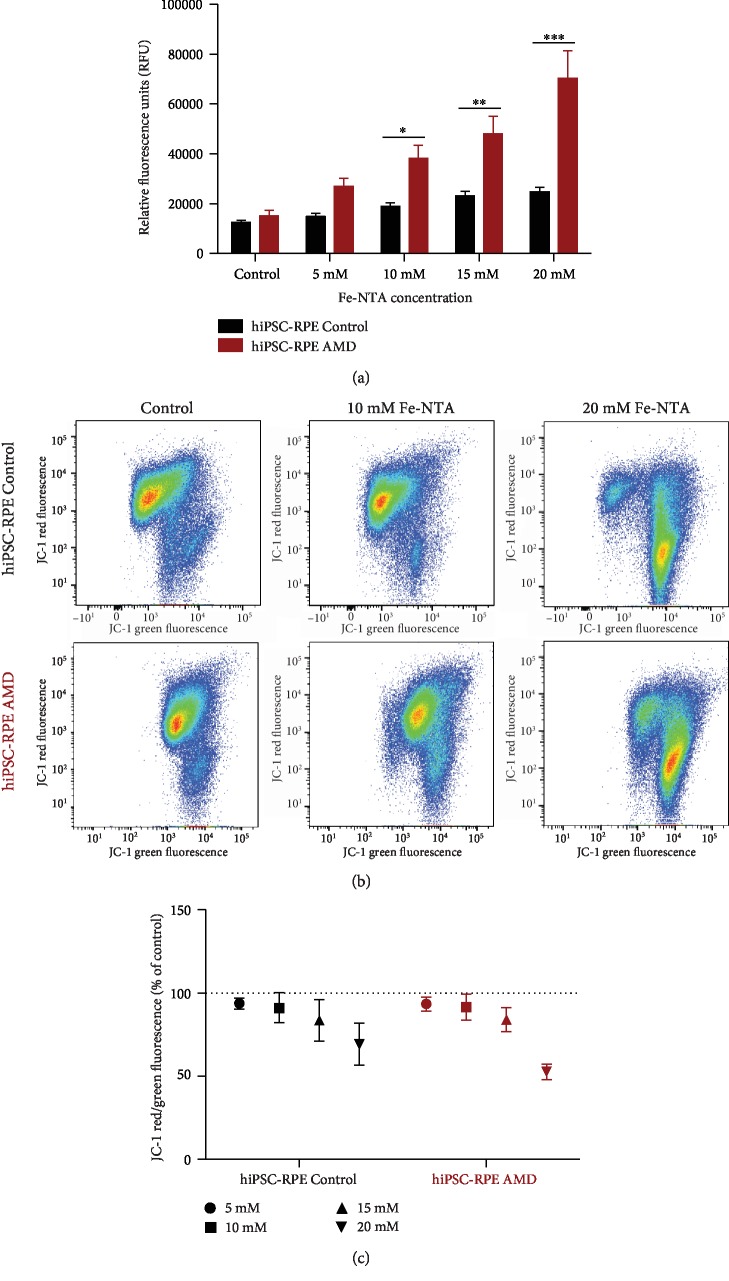
Analysis of oxidative stress production and mitochondrial function of hiPSC-RPE during Fe-NTA treatment. (a) Quantification of DCFH-DA oxidation induced by Fe-NTA treatment during 24 hours in both hiPSC-RPE Control (*N* = 6) and AMD (*N* = 6) cells (triplicate per cell line). (b) Flow cytometry analysis and (c) quantification of mitochondrial membrane potential using MitoProbe JC-1 staining. Control (*N* = 6) and AMD (*N* = 6) (two analyses per cell line). Statistical analysis: two-way ANOVA and post hoc Bonferroni test. ^∗^*p* < 0.05, ^∗∗^*p* < 0.01, and ^∗∗∗^*p* < 0.0001.

Fe-NTA treatment induced ROS production in a dose-dependent manner in both cell lines (Figure 4(a)). However, ROS production was significantly higher in hiPSC-RPE AMD cells compared to hiPSC-RPE Control cells from concentration of 10 mM (38248 RFU ± 5188 versus 182921 RFU ± 1413, *p* < 0.05) and above. ROS production is linked in part to Fenton reaction and lipofuscin cytotoxicity [32]. By electronic microscopy analysis, we observed that Fe-NTA treatment leads to melanolipofuscin production in both cell lines (Figure 3(c)).

### 3.5. Changes in Mitochondrial Potential Induced by Fe-NTA Are Similar in Both iPSC-RPE Cell Lines

Because mitochondria are responsible for most ROS production [31], the mitochondrial function in both basal and oxidative stress conditions was analyzed. The variation of mitochondrial potential was measured after Fe-NTA treatment in both cell lines by FACS using the MitoProbe JC-1 (Figure 4(b)). By quantification of the red/green fluorescence ratio, a dose-dependent decrease of the red fluorescence with Fe-NTA in both cell lines was measured. For 20 mM of Fe-NTA exposure, mitochondrial potential of hiPSC-RPE Control and AMD cells was decreased to 77.1% ± 9.63 and 67.8% ± 9.75, respectively (Figure 4(c)). No differences between hiPSC-RPE Control and AMD were observed for any dose of Fe-NTA.

### 3.6. hiPSC-RPE Control and AMD Cells Die by Apoptosis under Fe-NTA Exposure

**Figure 5:**
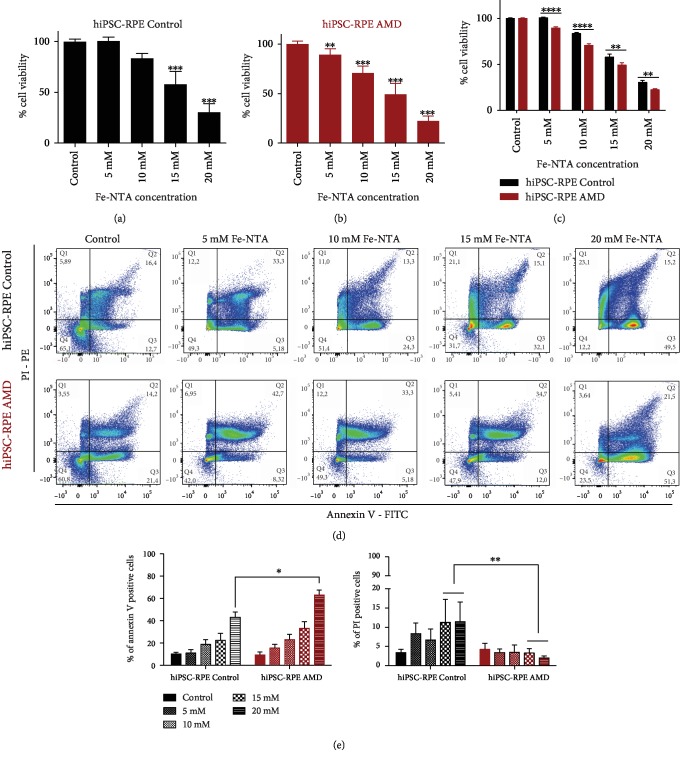
Analysis of cell death in both hiPSC-RPE Control and AMD cells during oxidative stress exposure. Quantification by an MTT test of cell death induced by Fe-NTA treatment during 24 hours in both (a–c) hiPSC-RPE Control (*N* = 6) and (b, c) AMD (*N* = 6) cells (triplicate per cell line). Statistical analysis: one-way ANOVA, post hoc Dunnett and two-way ANOVA, and post hoc Bonferroni. ^∗^*p* < 0.05, ^∗∗^*p* < 0.01, and ^∗∗∗^*p* < 0.0001. (c) Flow cytometry analysis and (b) quantification of cell death pathway using annexin-V PI staining. Control (*N* = 4) and AMD (*N* = 6) (one analysis per cell line). Comparison of each dose between hiPSC-RPE Control and AMD cells for each dose. Control cells (*N* = 6) and AMD cells (*N* = 6) (triplicate per cell line). Statistical analysis: two-way ANOVA and post hoc Bonferroni. ^∗^*p* < 0.05, ^∗∗^*p* < 0.01, and ^∗∗∗^*p* < 0.0001.

Cell death was not observed for Fe-NTA concentrations inferior to 10 mM in hiPSC-RPE Control cells (Figure 5(a)), while induced cell death of hiPSC-RPE AMD cells was observed at Fe-NTA concentrations equal or superior to 5 mM (Figure 5(b)). The cellular viability of hiPSC-RPE AMD cells was significantly lower than that of hiPSC-RPE Control cells from the concentration of 5 mM (89.3% ± 6 versus 100.5% ± 3.7, *p* < 0.001) and above (Figure 5(c)). Furthermore, 50% of cell survival in both hiPSC-RPE Control and hiPSC-RPE AMD cells was observed at 16.3 and 14.2 mM of Fe-NTA, respectively. By flow cytometry, we observed that hiPSC-RPE Control and AMD cells mainly die by apoptosis during Fe-NTA exposure (43% ± 4.8 and 62.9% ± 4.6 of annexin-V-positive cells, respectively); less than 10% of cells showed necrotic marker PI staining (Figures 5(d) and 5(e)).

### 3.7. Lysosomal Activity of hiPSC-RPE Control and AMD Cells under Oxidative Stress

**Figure 6:**
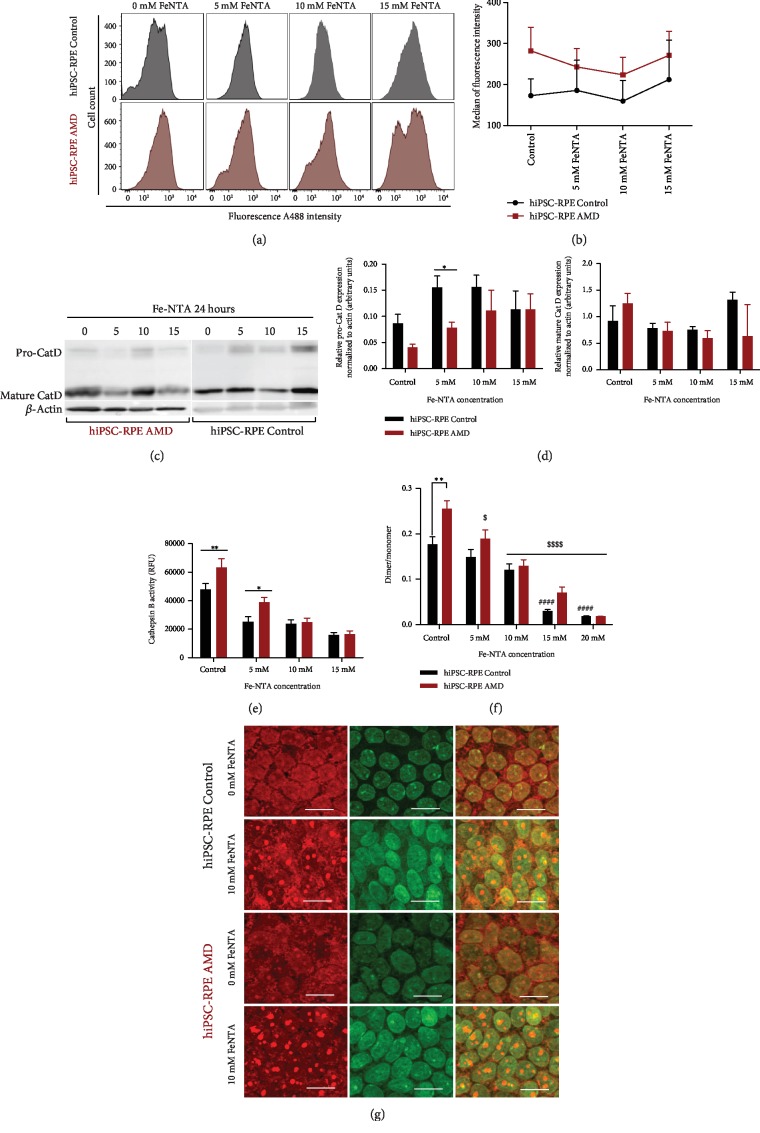
Analysis of hiPSC-RPE lysosomal activity during oxidative stress induced by Fe-NTA treatment. (a) Flow cytometry analysis and (b) quantification of global lysosomal activity in both hiPSC-RPE Control (*N* = 5) and AMD (*N* = 6) cells (one analysis per cell line). Statistical analysis: two-way ANOVA and post hoc Bonferroni. (c) Western blot analysis of pro-Cat D and mature Cat D and (d) western blot quantification in both hiPSC-RPE Control cells (*N* = 6) and AMD cells (*N* = 6) (one analysis per cell line). Statistical analysis: two-way ANOVA and post hoc Fisher LSD. Comparison of each dose between hiPSC-RPE Control and AMD cells (^∗^). ^∗^*p* < 0.05. (e) Analysis of hiPSC-RPE Control cells (*N* = 6) and AMD cells (*N* = 6) cell Cat B activity (four analyses per cell line). Statistical analysis: two-way ANOVA and post hoc Bonferroni. Comparison of each dose between hiPSC-RPE Control and AMD cells (^∗^). ^∗^*p* < 0.05 and ^∗∗^*p* < 0.01. (f) Comparison of the ratio of the dimer or oligomer to the monomer of acridine orange in hiPSC-RPE Control (*N* = 6) and AMD (*N* = 5) cells (triplicate per cell line). ^∗^Comparison of each dose between hiPSC-RPE Control and AMD cells for each dose. ^$^Comparison of each dose between basal condition for hiPSC-RPE Control and AMD cells. Statistical analysis: two-way ANOVA and post hoc Bonferroni. ^∗^^,$^*p* < 0.05, ^∗∗^^,$$^*p* < 0.01, and ^∗∗∗^^,$$$^*p* < 0.0001. (g) Lysosomal membrane stability was measured by AO staining under a fluorescence microscopy.

No significant difference in global lysosomal activity was observed in both hiPSC-RPE Control and AMD cells during basal condition and Fe-NTA exposure (Figures 6(a) and 6(b)). Next, we focus on Cathepsin D (Cat D), a major lysosomal enzyme that participated in the retinal homeostasis [33, 34]. Although the amount of mature Cat D protein was not apparently affected during Fe-NTA exposure, a dose-dependent increase of the amount of immature Cat D (pro-Cat D) in both hiPSC-RPE Control and AMD cells was observed (Figures 6(c) and 6(d)). For an exposure of 5 mM, this increase was significantly higher in hiPSC-RPE Control cells than in hiPSC-RPE AMD cells (Figure 6(d), *p* < 0.05). Cat D maturation is linked to Cat B activity: the intermediate form of Cat D is cleaved into a mature enzyme by this cysteine protease [34]. In basal conditions, Cat B activity was 1.3-fold higher in hiPSC-RPE AMD cells than in Control cells (47910 RFU (relative fluorescence unit) ± 4066 versus 63380 RFU ± 6110, *p* < 0.01) (Figure 6(e)). In both cell populations, a dose-dependent decrease of Cat B activity was observed during Fe-NTA treatment to 33% and 26% for 15 mM exposure in hiPSC-RPE Control and AMD cells, respectively (Figure 6(e)). Because lysosomal activity and Cat B-catalyzed hydrolysis are pH-dependent [35, 36], we measured lysosomal pH by AO (acridine orange) staining. As expected, the red/green fluorescence ratio of AO in hiPSC-RPE AMD cells indicated that the acidity of the vesicles was highest in this cell population in basal condition (0.177 ± 0.02 versus 0.255 ± 0.02, *p* < 0.01). Fe-NTA treatment leads to a dose-dependent decrease of red/green fluorescence in both cells (Figure 6(f)). Oxidative stress exposure leads to a lysosomal membrane permeabilization showed by an increase of green fluorescence compared to the basal condition without Fe-NTA (Figure 6(g)).

## 4. Discussion

AMD is the most common cause of legal blindness among persons over 60 years old [3]. In atrophic AMD, RPE cells are degenerating, secondarily leading to photoreceptor cell death [37]. The association with chorioretinal atrophy is unclear, but it is likely that the initial process of the disease is linked to RPE cells, because other pathological conditions with major choroidal thinning (i.e., degenerative myopia) lead to RPE atrophy. Since the last decade, many improvements in cell reprogramming and differentiation have been made to elucidate the pathological mechanisms leading to AMD. A deeper understanding of the mechanisms underlying atrophic AMD is necessary to evaluate promising new therapies.

In this study, 12 hiPSC lines, 6 from healthy individuals and 6 from patients suffering from atrophic AMD, were obtained and differentiated into hiPSC-RPE cells. hiPSC-RPE cells, derived from healthy or AMD patients, had characteristics similar to human RPE cells, with typical expression of RPE cell markers such as ZO-1, Pax6, Tyrosinase, and Bestrophin-1 and typical cobblestone epithelial morphology and pigmentation [38].

Classically, oxidative stress is minimized by antioxidant systems such as glutathione peroxidase or superoxide dismutase [39]. Aging is also associated with an increase of oxidative stress and ROS production [40]. Moreover, in the retina, the highest metabolic activity is located in the macula, with the highest ratio of RPE cells by a photoreceptor, leading to significant production of ROS in RPE cells during aging [41, 42]. However, with age, RPE cells lose the ability to compensate for ROS production by protective mechanisms, resulting in oxidative damage [39]. Therapies used to reduce the progression toward late forms of AMD consequently consist in diet supplementation with antioxidants [43]. In our study, we did not observe any differences in basal condition between hiPSC-RPE Control and AMD cells in terms of ROS level production. However, Fe-NTA treatment led to higher production of ROS in hiPSC-RPE AMD cells compared to that in hiPSC-RPE Control cells, suggesting that hiPSC-RPE AMD cells were more sensitive to the oxidative stress induced by Fe-NTA treatment than hiPSC-RPE Control cells. The higher production of ROS in hiPSC-RPE AMD cells may be explained by differences in damaged organite morphology leading to oxidative stress. Indeed, electronic microscopy analysis has shown that RPE cells obtained from postmortem AMD eyes contained more damaged mitochondria [23] and that the mitochondrial crista structure was disrupted [44]. Moreover, the overwhelming majority of cellular ROS has been tracked to the mitochondria [31]. In this context, mitochondrial activity was analyzed by potential JC-1 staining of the mitochondria membrane. Fe-NTA treatment led to alteration of the mitochondrial function without any difference between hiPSC-RPE Control and AMD cells. Mitochondria functional alteration alone could thus not explain the higher production of ROS during Fe-NTA treatment in hiPSC-RPE AMD cells. In fact, while mitochondrial alteration occurs physiologically with age, not all elderly people develop AMD [44]. Other mechanisms may contribute to overproduction of oxidative stress and to the RPE cell death.

Morphological analysis of postmortem AMD eyes highlighted distinct disease morphology of other RPE cellular components such as autophagosome and endoplasmic reticulum, which was wider and more irregular compared to healthy people [23]. In our electronic microscopy analysis, we did not observe organite morphological differences between hiPSC-RPE Control and AMD cells in basal condition and after Fe-NTA treatment, while the latter led to lipofuscin production in both cell lines. Endoplasmic reticulum stress could induce VEGF secretion, which could be involved in the lysosomal accumulation of lipofuscin within RPE cells [45]. The accumulation of lipofuscin [15] in senescent RPE cells is often considered a hallmark of aging [46]. As previously described [47], aging is the major risk factor of AMD development. Morphological changes of RPE cells related to aging include loss of melanin granules, increased density of residual body, and accumulation of lipofuscin [16]. Here, we have demonstrated that both hiPSC-RPE Control and AMD cells derived from aged individuals express a senescent profile by the expression of SA-*β*-Gal activity, a classical aging marker [30]. Mitochondrial morphology modifications related to senescence confirmed the aging phenotype of hiPSC-RPE Control and AMD cells achieved in this study. The increase of *β*-Gal activity with replicative senescence and aging is linked to increased lysosomal activity [48]. Indeed, *β*-Gal activity may be the reflection of lysosomal activity [46]. Lysosomal activity is a process involved in the degradation of photoreceptor outer segments in RPE cells [49] and in replacement of damaged mitochondria by mitophagy [50]. With aging, degradation and/or reparation processes could be less efficient and may lead to an accumulation of damaged components such as mitochondria, as previously shown [23]. Furthermore, the accumulation of iron in AMD is associated with decreased lysosomal activity [22, 51]. One of mechanistic AMD hypotheses is based on lipid peroxidation related to impairment of lysosomal function. This process may contribute to RPE cell dysfunction via intracellular processes that directly reduced the lysosomal activity [22]. In our study, we did not observed any difference in global lysosomal activity between hiPSC-RPE Control and AMD cells, neither in basal nor in oxidative stress condition. However, we observed that Cat B activity was higher in hiPSC-RPE AMD cells and probably linked to a more acidic lysosomal environment. Indeed, Cat B cleaved the N-terminal segment of Cat D leading to its activation at low pH [52]. In our study, we also observed that, during Fe-NTA exposure, Cat B activity is inhibited in the two populations. This result is correlated with the dose-dependent increase of the immature form during oxidative stress condition, which was observed in both populations, while appearing more rapidly in hiPSC-RPE Control cells. The lower Cat B activity in hiPSC-RPE Control cells could explain that more pro-Cat D was found in this population compared to hiPSC-RPE AMD cells. As previously described with an immortalized cell model of RPE, ARPE-19, iron accumulation increases the immature form of Cat D, without any change in the mature form [16]. Our study also confirmed these results in hiPSC-RPE Control and AMD cells. Any abnormalities in this enzyme system may have pathological consequences. Indeed, the presence of Cat D has been shown to be associated with accumulation of undigested (photoreceptor outer segment) derived debris, which could contribute to drusen formation. Chen et al. [16] hypothesized that iron overload in ARPE-19 inhibited the conversion process of pro-Cat D into Cat D.

In our study, we compared hiPSC-RPE cells derived from somatic cells of healthy persons to those derived from patients affected by atrophic AMD. Taken together, our results demonstrate that hiPSC-RPE cells from patients affected by atrophic AMD had a specific phenotype compared to hiPSC-RPE Control cells. Recently, Golestaneh et al. [4] obtained the same typical phenotype with hiPSC-RPE cells derived from postmortem RPE cells as the control population in response to oxidative stress induced by H_2_O_2_, which is currently used *in vitro* to rapidly induce oxidative stress. In our study, treatment with iron was preferred as a mean of inducing iron accumulation as it mimics the prooxidant microenvironment observed in AMD and during the aging process. Iron is a natural element that accumulates with normal aging [16]. Additionally, it has been observed that concentration of iron was greater in the macular area and RPE cells in individuals affected by atrophic AMD [21]. Specific iron accumulation has been associated in RPE cells and photoreceptor degeneration in other retinal injuries [20]. Oxidative stress induced by iron is responsible for specific activation pathways such as ferroptosis [53]. Taken all together, these different studies suggested that iron is probably a more reliable tool than H_2_O_2_ (i) to induce oxidative stress *in vitro* and (ii) to model the oxidative stress mechanisms that occur *in vivo* in the RPE cells of AMD patients.

**Figure 7:**
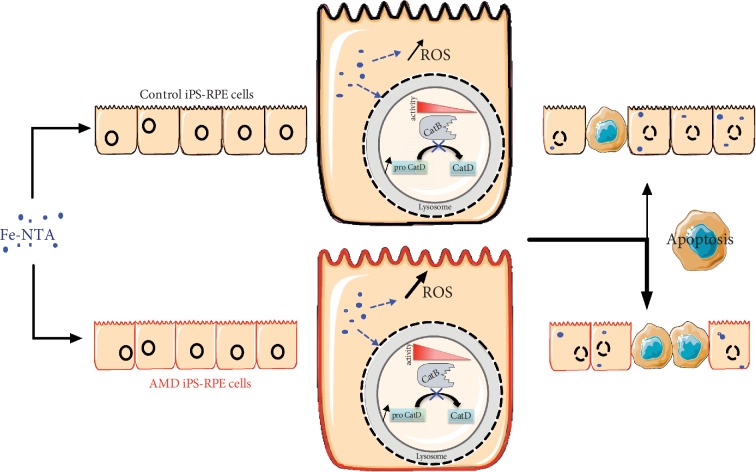
Graphic summary of typical disease phenotype of hiPSC-RPE AMD cells compared to hiPSC-RPE Control cells in the production of reactive oxygen species, cellular viability, and lysosomal function in response to oxidative stress induced by intracellular accumulation of iron.

We demonstrated that hiPSC-RPE cells derived from patients affected by atrophic AMD expressed a specific disease phenotype compared to iPSC-RPE cells derived from healthy individuals as illustrated in Figure 7. Indeed, hiPSC-RPE AMD cells were more sensitive to oxidative stress and produced more ROS under a prooxidant environment. They also show dysregulation of lysosomal activities in basal condition and during oxidative stress exposure. In our study, we demonstrate that, despite reprogramming and differentiation process, cells derived from AMD patients exhibit pathological mechanisms leading to a distinct phenotype. These results support the evidence that (i) lysosomal activity of RPE cells plays a central role in the pathogenesis of AMD and (ii) hiPSC-RPE cells derived from erythroblasts of AMD patients express a disease phenotype and (iii) could be a useful tool to study pathological mechanisms leading to the development of AMD or to evaluate potential therapeutic molecules.

## 5. Conclusion

In this study, hiPSC-RPE cell lines derived from atrophic AMD patients were compared to hiPSC-RPE cell lines derived from healthy people. We observed a critical difference in terms of production of reactive oxygen species, cellular viability, and lysosomal function in response to oxidative stress induced by intracellular accumulation of iron. These findings demonstrate that (i) hiPSC-RPE AMD cells have a typical disease phenotype compared to hiPSC-RPE Control cells and that (ii) dysfunction of RPE cell lysosomal activity is involved in pathological mechanisms of AMD.

## Data Availability

The datasets generated and/or analyzed during the current study are available from the corresponding author on reasonable request.
