# Corrigendum to “Cathepsin B pH-Dependent Activity Is Involved in Lysosomal Dysregulation in Atrophic Age-Related Macular Degeneration”

**DOI:** 10.1155/2020/3510764

**Published:** 2020-07-17

**Authors:** Audrey Voisin, Christelle Monville, Alexandra Plancheron, Emile Béré, Afsaneh Gaillard, Nicolas Leveziel

**Affiliations:** ^1^University of Poitiers, Laboratoire de Neurosciences Expérimentales et Cliniques, Equipe Thérapie Cellulaire dans les Pathologies Cérébrales, Poitiers F-86073, France; ^2^INSERM, U1084, Laboratoire de Neurosciences Expérimentales et Cliniques, Equipe Thérapie Cellulaire dans les Pathologies Cérébrales, Poitiers F-86022, France; ^3^CHU Poitiers, Poitiers F-86021, France; ^4^INSERM, UMR861, I-Stem, AFM, Genopole Campus 1 Evry F-91030, France; ^5^UEVE-Paris Saclay, UMR861, I-Stem, AFM, CRCT, Corbeil-Essonnes F-91100, France; ^6^CECS/I-Stem AFM, CRCT, Corbeil-Essonnes F-91100, France; ^7^Plateforme ImageUP, 1 Rue Georges Bonnet, F-86022 Poitiers, France; ^8^Vision and Eye Research Institute, Cambridge, UK

In the article titled “Cathepsin B pH-dependent activity is involved in lysosomal dysregulation in atrophic age-related macular degeneration” [1], an affiliation was omitted in error. This affiliation has been added to the affiliation list above as number “8,” and the author affiliations have been corrected.
